# Supplementary material for: Coordinated regulation of Mdr1- and Cdr1-mediated protection from antifungals by the Mrr1 transcription factor in emerging Candida spp
Source: bioRxiv. 2025 May 5:2025.05.04.652153. Preprint. [Version 1] doi: 10.1101/2025.05.04.652153 (PMC12248020; doi:10.1101/2025.05.04.652153)
Supplement: Supplement 1 [file NIHPP2025.05.04.652153v1-supplement-1.pdf]

Figure S1: Consensus Mrr1-binding DNA motif in the promoter regions of Mrr1 targets. (A) The positions of the 14-nt (orange hatches) and 9-nt Mrr1-binding motifs (consensus Mrr1-binding motif or cMBM; blue hatches) in the ~890 bp upstream intergenic regions of *MDR1* in *C. lusitaniae* L17 *and* ATCC 42720 strains. (B) cMBM location in the 1 kb upstream intergenic regions of *CDR1* from *C. lusitaniae* L17 *and* ATCC 42720. (C) cMBM location in the 1 kb upstream intergenic regions of the *CDR1* homologs of *C. parapsilosis* CDC317, *C. auris* B8441*, C. albicans* SC5314 and *C. lusitaniae* ATCC 42720. The phylogenetic tree was constructed using the *CDR1* nucleotide sequences. (B, C) The intergenic region upstream of *CDR1* in ATCC 42720 is 326 bp. Grey arrow indicates the adjacent ORF *CLUG_03114.*

Figure S2: Comparison of binding profiles of constitutively active and low-activity Mrr1. (A-C) Comparison of data from Figures 1D-F and Figures 5A-C to highlight the similarities in peak profiles. HF-Mrr1^{Y813C} (in blue) and HF-Mrr1^{ancestral} (in grey) CUT&RUN read coverage plots normalized per 20 bp bin size. Chromosomal positions of regions containing *MDR1, CDR1* and *FLU1* and adjacent genes are represented to scale with boxes and arrows. Peaks from HF-Mrr1-bound DNA recovered by an α-FLAG antibody and for the non-specific binding control recovered via IgG are shown. Signal indicates the average read density in α-FLAG relative to IgG within the peak region.

Figure S3: Biochemical and phenotypic analysis of HF-tagged Mrr1 variants. (A) Western blot of whole cell protein lysates of U04 strains expressing N-terminal 6xHis-3xFLAG-tagged Mrr1 (HF-Mrr1) variants. HF-Mrr1 was probed using an α-FLAG antibody.

bioRxiv preprint doi: https://doi.org/10.1101/2025.05.04.652153; this version posted May 6, 2025. The copyright holder for this preprint (which was not certified by peer review) is the author/funder, who has granted bioRxiv a license to display the preprint in perpetuity. It is made available under aCC-BY 4.0 International license.

Mean ± SD of HF-Mrr1 band intensities normalized to total protein (n= 4 biological replicates). (B) FLZ MIC of U04 clinical isolate (native allele *MRR1^{Y813C}*) and U04 *mrr1*Δ complemented with untagged or *HF-MRR1* was determined by broth microdilution assays. The data shown represent the mean ± SD from three independent experiments. There were no significant differences observed between data from strains with untagged Mrr1 variants and data from strains with their respective HF-tagged counterparts. Strains with constitutive Mrr1 activity are in bold.

Figure S4: Global binding profiles of constitutively active and low-activity Mrr1. Circos plot showing global CUT&RUN-determined Mrr1-binding peaks of HF-Mrr1^{Y813C} (in blue), HF-Mrr1^{ancestral} (in grey) and HF-Mrr1^{L1Q1*} (in orange) in the *C. lusitaniae* L17 genome. Mrr1-binding peaks with a signal ≥2-fold compared to their respective IgG backgrounds and up to 1 kb away from the nearest ORF from Experiment 1 (see Supplemental Files 1, 4 and 5) were used. The genomic positions of the 25 differentially expressed genes that constitute the Mrr1-regulon are marked with the L17 gene IDs.
